# BNST specific mGlu5 receptor knockdown regulates sex-dependent expression of negative affect produced by adolescent ethanol exposure and adult stress

**DOI:** 10.1038/s41398-021-01285-y

**Published:** 2021-03-17

**Authors:** Chelsea R. Kasten, Eleanor B. Holmgren, Mollie R. Lerner, Tiffany A. Wills

**Affiliations:** 1 Department of Cell Biology and Anatomy, LSU Health Sciences Center New Orleans, New Orleans, LA USA; 2 Neuroscience Center of Excellence, LSU Health Sciences Center New Orleans, New Orleans, LA USA

**Keywords:** Molecular neuroscience, Genetics

## Abstract

Adolescent alcohol use is one of the strongest predictors for the development of an alcohol use disorder (AUD). Notably, this period of risk coincides with the development of affective disorders, which disproportionately impact and drive problematic drinking behavior in women. Stress is a particularly salient factor that drives relapse during periods of abstinence. Previous work in our lab has shown that adolescent intermittent ethanol vapor (AIE) produces sex-dependent changes in glutamatergic activity in the bed nucleus of the stria terminalis (BNST) and behavioral outcomes following acute restraint stress in adulthood. In females, AIE disrupts group 1 metabotropic glutamate (mGlu1/5) receptor activity and enhances anhedonia-like behavior. The current study site-specifically knocked down mGlu5 receptors in the BNST of male and female *Grm5*^loxp^ mice, exposed them to AIE, and observed the interaction of AIE and stress on negative affect-like behaviors in adulthood. These negative affect-like behaviors included the novelty-induced hypophagia task following acute restraint stress, open field activity, and contextual fear conditioning. Overall, we replicated our previous findings that AIE enhanced anhedonia-like activity in the novelty-induced hypophagia task in females and fear acquisition in males. The primary effect of BNST-mGlu5 receptor knockdown was that it independently enhanced anhedonia-like activity in females. Correlation analyses revealed that behavior in these paradigms showed poor interdependence. These results indicate that preclinical models of negative affective-like states encompass distinct features that may have independent, clinically relevant mechanisms. Further, modulating mGlu5 receptors is a prospective treatment target for females experiencing anhedonic-like states that make them susceptible to alcohol relapse.

## Introduction

Adolescent alcohol use is known as one of the strongest predictors for the development of an alcohol use disorder (AUD)^1^, due in part to persistent neuroadaptations that may follow repeated use^2,3^. Alcohol use during this critical time period might increase the risk of developing a stress or anxiety disorder, which are more prevalent in females^4,5^. These disorders fall under the umbrella of “negative affect”; the emotional states that underlie numerous psychiatric disorders and are known to drive excessive alcohol drinking, particularly in women^6–8^. Therefore, adolescent alcohol exposure may produce long-term sex-dependent expression of emotional dysregulation that influences problematic drinking in women.

Many facets of negative affect, such as anxiety, depression, and hypervigilance, can be assessed using preclinical models. A recent review from the Neurobiology of Adolescent Drinking in Adulthood (NADIA) Consortium indicates that adolescent alcohol exposure generally produces long-lasting changes in anxiety-like activity in adulthood in rodents^9^. Adolescent alcohol exposure has also been linked to alterations in despair/depressive-like behavior in adulthood that may be sex-dependent, but these results are not consistent^10–16^. The NADIA Consortium notes that these inconsistencies may be due to the alcohol exposure paradigm and that potential differences between sexes have not been systematically addressed^9^. Further, many studies utilize a limited number of behavioral tasks to observe negative affect-like states, even though negative affect is a multi-dimensional concept^17–19^. Recent work from our lab has demonstrated that adolescent alcohol intermittent ethanol vapor (AIE) results in sex-dependent expression of stress-induced negative affect-like states in adulthood. AIE did not alter basal anxiety- or anhedonic-like activity in the absence of stress. However, AIE-exposed female mice showed enhanced anhedonia-like activity in the novelty-induced hypophagia (NIH) task following restraint stress, whereas AIE-exposed male mice showed increased low-intensity foot shock-induced freezing^20^.

One region known to regulate mood-related behaviors is the sexually dimorphic bed nucleus of the stria terminalis (BNST)^21^. The BNST is a site of convergence for stress- and alcohol-related information^7^, which specifically mediate glutamatergic transmission^22–24^. Notably, AIE produces sex-dependent changes in glutamatergic transmission in the BNST of C57Bl/6J mice during acute withdrawal and in adulthood following stress. In male mice, these signaling changes are mediated by the GluN2B-containing NMDA receptors^25^, whereas in female mice they are mediated by group 1 metabotropic glutamate (mGlu1/5) receptors^20^.

Recent work in our lab has demonstrated that this disrupted mGlu1/5-mediated signaling in the BNST is associated with stress-induced anhedonic states in adulthood following AIE that are present in female, but not male, mice^20^. Preclinical studies have consistently implicated mGlu5 receptors in reducing alcohol intake and regulating alcohol-induced negative affect-like behavior across a diverse array of behavioral paradigms, including despair- and anxiety-like behaviors^26^. Further, human postmortem studies show sexually dimorphic mGlu5 receptor expression in individuals with major depressive disorder (MDD) compared to control samples. In the dorsolateral prefrontal cortex, female MDD patient tissue had increased mGlu5 expression, whereas male MDD patient tissue had decreased mGlu5 expression^27^. However, increased mGlu5 expression was observed in locus coeruleus neurons of male subjects with MDD^28^.

mGlu5 receptors may also serve as a biomarker for AUDs and interventions. Genetic variants in the *mGluR-eEf2*-AMPAR pathway, including *GRM5*, predict alcohol intake in community-based samples^29^. In healthy, low-drinking participants, mGlu5 receptor availability is positively associated with “feeling high” in response to alcohol^30^. Alcohol-dependent individuals show reduced mGlu5 receptor availability that widely recovers over 6 months of abstinence^31,32^, although smoking status may be a contributing variable^33^. Notably, mGlu5 availability is positively correlated with craving alcohol as a negative reinforcer and relapse in alcohol-dependent individuals, but recovery of mGlu5 availability over time is associated with reduced alcohol craving^31–33^. Beyond serving as a biomarker, one enticing aspect of investigating mGlu5 receptors is that they represent a clinically viable target. Current potential therapies have shown minimal adverse outcomes over relatively short-term treatment periods^34–36^, acute alcohol does not affect compound metabolism^37^, and a number of clinical trials have recently wrapped up or are currently recruiting participants to observe alcohol-associated outcomes^38–41^.

The current study sought to identify the role of BNST-specific regulation of mGlu5 receptors in negative affective-like behaviors. Using a Cre-lox strategy for the mGlu5 receptor gene, mGlu5 receptors were site-specifically knocked down in the dorsolateral BNST (dlBNST) of male and female mice. The mice were then exposed to AIE and allowed to age into adulthood, at which time anhedonia-like activity in the NIH task following restraint stress, basal anxiety-like activity in the open field, and foot shock-induced freezing were observed. We hypothesized that the AIE GFP-control mice would show the previously observed sex-dependent phenotypes^20^, and that mGlu5 receptor knockdown in the BNST would modulate this behavior in female mice. Elucidating the role of mGlu5 receptors in these behavioral phenotypes may lead to more effective approaches for treating AUDs and comorbid, sex-dependent negative affect-like symptomology.

## Methods

### Mice

*Grm5*^loxp^ breeder pairs were generously provided by Dr. Danny Winder (Vanderbilt University, Nashville, TN; Jackson Laboratories, Bar Harbor, ME: B6.129-*Grm5*^*tm1.1Jixu*^/J) and bred in-house. Mice were weaned at PND21 and housed separately with littermates (2–5 per cage). Food and water were provided ad libitum. All procedures were approved by the Animal Care and Use Committee at Louisiana State University Health Sciences Center and followed the guidelines set forth by the National Institutes of Health^42^.

### Stereotaxic surgery

A total of 95 mice (51 females, 44 males) received intracranial surgery targeting the dlBNST (AP: +0.14, L: ±0.8, D: −4.14) between PND22-29. The surgery protocol was modified from Moore and Boehm^43^. Briefly, mice were anesthetized using a ketamine/xylazine cocktail (1 ml of 100 mg/ml ketamine, 0.1 ml of 100 mg/ml xylazine, and 8.9 ml sterile saline) at a volume of 0.1 ml/10 g of weight. Mice were then placed on an Angle Two Small Animal Stereotaxic Instrument (Leica Microsystems, Wetzlar, Germany). rAAV5/CamkII-GFP-Cre (cre) or rAAV5-CamKIIa-eGFP (GFP; control) were obtained from the Gene Therapy Center Virus Vector Core Facility (The University of North Carolina at Chapel Hill, Chapel Hill, North Carolina). The virus was infused at a rate of 100 nl/min for a total volume of 200 nl/side. Injectors were left in place for 5 min to allow time for the virus to diffuse from the tips. At least one mouse/cage received cre or control virus to reduce potential littermate confounds. Mice received 5 g/kg ketoprofen immediately, 24 h, and 48 h post-surgery and recovered for at least 4 d prior to vapor exposure. Surgeries for each cohort were completed within a 36-h window.

### Western blot

Viral knockdown was confirmed using western blot for mGlu5 receptors (1:3000; AB5675; Millipore Sigma), normalized to GAPDH signal (1:10,000; AB8245; Abcam, Cambridge, MA), in 500-µm thick dlBNST-containing punches from AIE-naïve adult male and female mice as previously described^20^. Mice were infused with virus during adolescence.

### Ethanol vapor exposure

Six mice died following surgery, leaving 48 females and 41 males to be run through vapor exposure and subsequent behavioral testing. Cages were randomized and assigned to air or ethanol exposure based on sex, vapor cohort, and parents. When possible, at least one cage/sex was run through air and ethanol in each cohort. Male and female mice were exposed in separate chambers that can be independently controlled to produce the same BAC in each sex. The ethanol vapor chambers were maintained to achieve BACs of ~200 mg/dL, which were monitored using non-experimental mice. Vapor exposure began between PND27-35 following previously published protocols^25^. Briefly, mice were placed into the chamber in their home cages overnight for two, 4-d cycles of 16 h vapor exposure. Mice were placed back in the vivarium for the 8 h out-of-chamber. The 4-d cycles were separated by 3 d.

### Behavioral tasks

Mice were aged undisturbed until adulthood (PND70 + ). Most mice were run through all behavioral tasks (see Supplemental Table 1 for details).

#### NIH and acute restraint stress

Procedures were modified from previously published protocols^44,45^. Mice were given 2 h access to Ensure vanilla-flavored nutritional shake in their home cage in a dimly lit room (~70 lux) under a hood. Latency to consume Ensure was recorded on the second day of training; mice that failed to consume were run through the test portion but excluded from further NIH analyses. On the test day, mice were restrained in well-ventilated 50-mL conical tubes and left undisturbed for 1 h^46^ before being returned to their home cage for 1 h. Mice were then individually placed into empty standardized mouse cages under the hood with lights on (~300 lux) and given 45 min to consume Ensure. The testing sessions were video recorded and two individuals blind to treatment condition scored the latency to initiate consumption on the test day.

#### Open field activity

Three to four weeks later, mice (PND91+) were exposed to the open field arena (TruScan mouse arena, Coulbourn Instruments, 25 × 25 cm) of the contextual fear apparatus located in a sound-attenuated chamber for 3 min prior to shock administration. This 3 min period was used to assess basal anxiety-like activity, quantified as the % of total distance traveled in the center of the chamber.

#### Contextual fear conditioning

Procedures were modified from a previously published protocol^46^. Following the 3 min habituation period, mice were exposed to six shocks delivered on a 30-s variable inter-stimulus interval through the rod shock floor connected to the TruScan Photobeam LINC system. Shocks were 0.4 mA, 1 s duration for male mice and 0.3 mA, 500 ms for female mice. Our lab has previously validated these intensities as producing similar, low levels of freezing in C57Bl6/J mice that allow for the detection of heightened fear responses in AIE-exposed animals^20^. Approximately 24 h post-training, mice were re-exposed to the chamber for a 15 min extinction session during which no shocks were administered. Data measurements were collected using Tru Scan software on a Windows computer.

### Histological confirmation

Following behavior, mice were initially perfused using 4% PFA under ketamine/xylazine and targets were checked in 100 µm slices. To achieve the best visualization, we switched to sacrificing the mice and using live slices (200–300 µm) for ex vivo imaging^25^. The infusion region was visualized by exciting GFP at 488 nm using a BXWI51 microscope (Olympus). Animals were considered hits when a majority of the fluorescence was located within the dlBNST. Three mice (1 male air-cre, 1 female air-cre, 1 female air-GFP) were removed due to off-target surgeries.

### Statistical analysis

Only mice with injections determined to target the dlBNST were included in statistical analyses. (Total female mice analyzed: 11 air-GFP, 12 air-cre, 12 AIE-GFP, 11 AIE-cre. Total male mice analyzed: 11 air-GFP, 9 air-cre, 11 AIE-GFP, 9 air-cre. Target group sizes were determined by previous findings^20^). Male and female mice were analyzed separately due to sex-dependent AIE-induced changes in these behavioral tasks^20^. All data were checked for normality and GraphPad Prism software (ver. 8.3) was used for analyses. Two-way ANOVAs assessing vapor history*viral status were run for the NIH and open field tasks. A three-way ANOVA assessing time point*vapor history*viral status was run for the contextual fear paradigm, as well as two-way ANOVAs within each time point. All values are presented as means ± SEM with *p* ≤ 0.05 considered significant (two-tailed), adjusted for post-hoc analyses. The overall analyses were run once, combining data from 16 litters of mice run in seven distinct cohorts. Sidak’s post-hoc tests comparing air- vs. AIE-history within viral group were run to confirm replication of the baseline phenotypes previously observed in our lab^20^.

## Results

### Cre infusion significantly knocks down mGlu5 receptors in the dlBNST

**Fig. 1:**
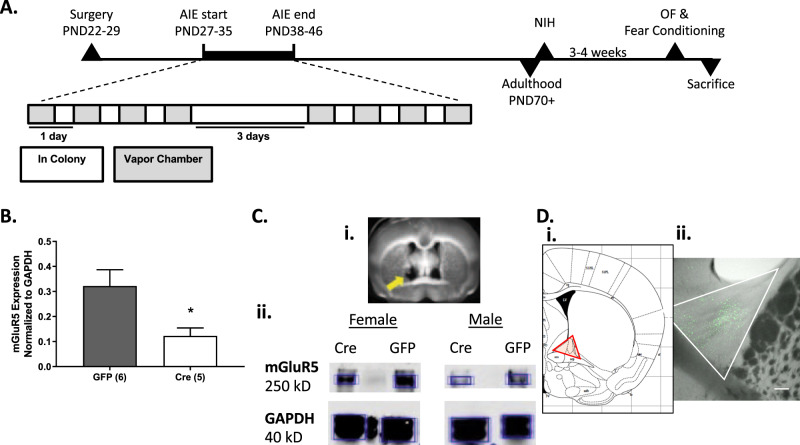
Cre infusion knocks down mGlu5 receptors in the dlBNST. Experimental timeline (**A**). Cre virus infusion induced significant mGlu5 receptor knockdown in the dlBNST using western blot analysis of BNST tissue punches (**B**). Representative image of punch location indicated by arrow (Ci), representative western blots (Cii), and viral expression in the dlBNST (**D**). Female and male samples were run on separate blots. Cropping is indicated by surrounding white space (Cii). Panel (Di) was modified from Paxinos & Franklin^79^ coronal diagram Bregma 0.26 and highlighted region corresponding to the image in Dii. Dii was created by merging a bright-field image (color, merge channel: gray) and GFP image (color, merge channel: green) of the same image field using ImageJ. Scale bar = 100 μm. Data shown as mean ± SEM, ns = 5–6. *p < 0.05.

The study timeline is shown in Fig. 1A. To validate the flox/cre strategy, mice were infused with cre or control virus at the adolescent timepoint and allowed to age into adulthood without vapor exposure. A t-test indicated that cre infusion targeting the dlBNST significantly reduced mGlu5 receptor expression normalized to GAPDH (*p* < 0.05, Fig. 1B, C), which could be visualized by optical excitation of the GFP tag (Fig. 1D).

### dlBNST mGlu5 receptor knockdown increases anhedonic-like behavior in female mice

**Fig. 2:**
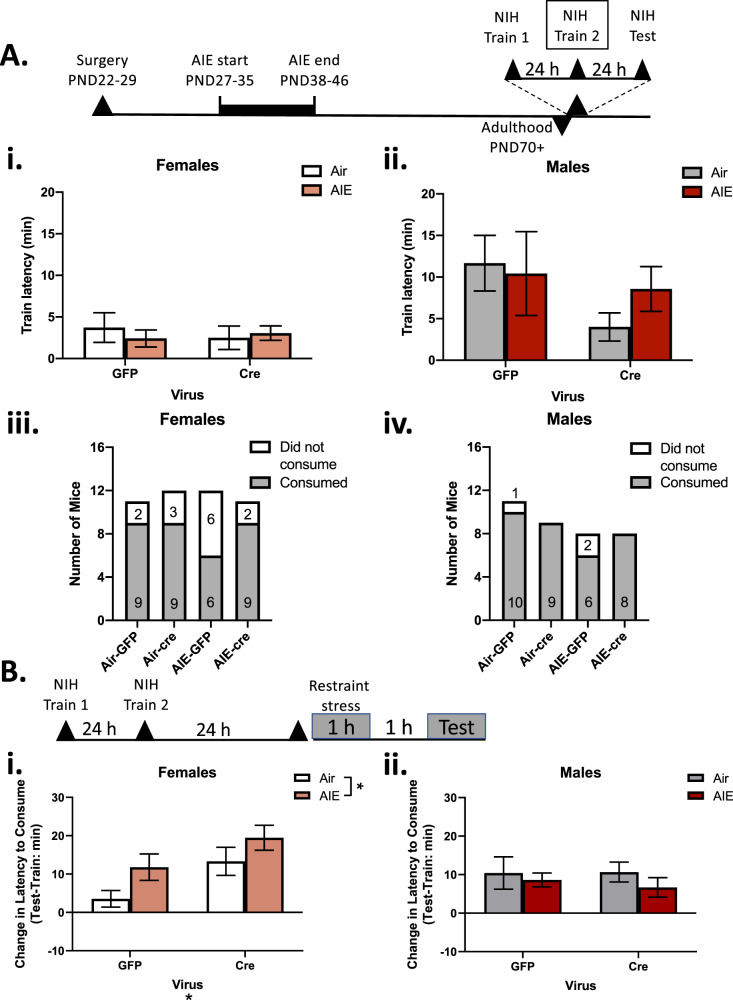
Anhedonic-like behavior in dlBNST mGlu5 receptor knockdown mice. NIH timeline (**A**). Latency to consume an appetitive reinforcer in the home cage on the second day of training in female (i) and male (ii) mice. Data shown as mean ± SEM, ns = 11–12 for female mice and 8–11 for male mice. The proportion of female (iii) and male (iv) mice that consumed on the second day of training. Data shown as absolute numbers. NIH test day timeline (**B**). Change in latency to consume an appetitive reinforcer in an aversive environment following restraint stress in female (i) and male (ii) mice. Data shown as mean ± SEM, ns = 6–9 for female mice and 6–10 for male mice. Main effect at *p < 0.05.

The NIH task is a preclinical analogue of negative affect-associated despair and anhedonia. Restraint stress uncovers enhanced anhedonic-like behavior in AIE-exposed female, but not male, mice^20^. Further, these stress- and AIE-induced changes in NIH behavior can be modulated by systemic treatment with the mGlu5 receptor antagonist MTEP^20^. To test the role of BNST-specific mGlu5 receptors on this behavior, we site-specifically knocked down these receptors in the BNST. Mice were exposed to AIE, then restraint stress-induced changes in the NIH task were observed in adulthood (Fig. 2A, B).

On the second training day, prior to restraint stress, the latency to consume was measured in the home cage. A vapor history*viral status ANOVA revealed no significant interaction or main effects on latency to consume in female mice (*p*’s > 0.05; Fig. 2Ai). In male mice, a vapor history*viral status ANOVA revealed no significant interaction or main effects on latency to consume (*p*’s > 0.05; Fig. 2Aii), although there was a notable amount of variance in the GFP-infused group. Further, a chi-square test revealed that a significantly higher proportion of female mice failed to consume on the training day (13/46, 28.3%) compared to male mice (3/35, 8.6%) [*χ*^2^(1, *N* = 81) = 4.861, *p* < 0.05; Fig. 2Aiii, iv). These mice were run through the test day but were not included in the test day analyses.

**Fig. 3:**
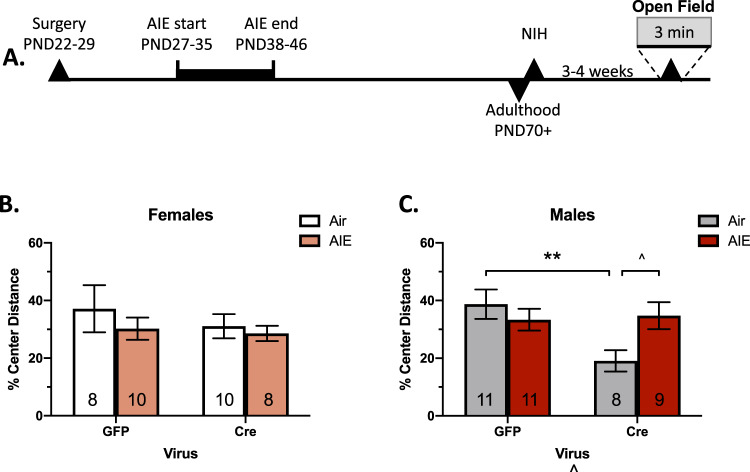
Basal anxiety-like activity in dlBNST mGlu5 receptor knockdown mice. Open field timeline (**A**). Percent of total distance traveled in the center of the open field in female (**B**) and male (**C**) mice. Data shown as mean ± SEM, ns = 8–10 for female mice and 8–11 for male mice. **p < 0.05, ^*p* < 0.052.

To account for individual and intra-sex variability in consummatory latencies^5,47^, latencies on the test day were quantified as the change in consummatory latency from the training day^20^. In female mice, there was a significant main effect of vapor history [*F*(1,29) = 4.97, *p* < 0.05] and virus [*F*(1,29) = 7.27, *p* < 0.05] (Fig. 3Bi) but no interaction of vapor history*viral status on change in latency to consume on the test day following restraint stress (*p* > 0.05). AIE history and BNST mGlu5 receptor knockdown independently increased latency to consume under aversive conditions. Post-hoc tests revealed no significant effect of AIE-history within virus groups (Sidak *p*’s > 0.05). In male mice, we observed no significant interaction or main effects of vapor history and virus following restraint stress in the NIH task (*p*’s > 0.05, Fig. 3Bii). Overall, these results replicate our previous finding that restraint stress induces an anhedonic-like phenotype in adulthood in female mice with an AIE history^20^. Further, mGlu5 knockdown in the BNST enhances this anhedonic-like state in female, but not male mice.

### dlBNST mGlu5 receptor knockdown enhances basal anxiety-like activity in males

Distance traveled in the center of the open field is a well-established model of anxiety-like activity^48^. In female mice, a vapor history*viral status ANOVA revealed no significant interaction or main effects (*p*’s > 0.05) on % of distance traveled in the center of the open field of the fear chamber in the 3 min prior to shock (Fig. 3A, B).

In male mice, a vapor history*viral status ANOVA revealed no significant effect of vapor history (*p* > 0.05), but a main effect of the virus [*F*(1,35) = 4.12, *p* = 0.05] and a significant interaction [*F*(1,31) = 5.47, *p* < 0.05] (Fig. 3C). Post-hoc analysis indicated that the effect of the virus was driven by the air-history group with mGlu5 receptor knockdown, with a strong trend towards a significant difference between air-cre and AIE-cre mice (Sidak’s; GFP: *p* > 0.05, cre: *p* = 0.052). Regardless of this, air-cre mice showed significantly greater anxiety-like activity than their air-GFP counterparts (*p* < 0.01).

### dlBNST mGlu5 receptor knockdown does not alter contextual fear acquisition in either sex but disrupts extinction in female mice

**Fig. 4:**
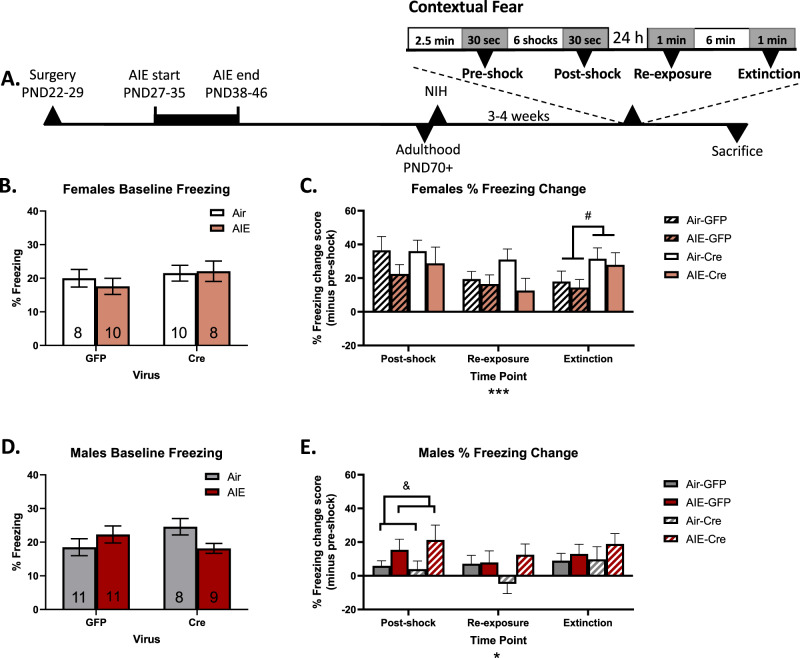
Contextual fear acquisition, retention, and extinction in dlBNST mGlu5 receptor knockdown mice. Contextual fear timeline (**A**). Pre-shock freezing levels in female (**B**) and male (**D**) mice. Freezing levels across the task adjusted for pre-shock freezing levels in female (**C**) and male (**E**) mice, where 0 indicates no change from pre-shock freezing. Data shown as mean ± SEM, ns = 8–10 for female mice and 8–11 for male mice. Omnibus main effect at *p < 0.05 and ***p < 0.001. Within time point main effect of virus at #*p* < 0.05 or main effect of vapor at &*p* < 0.05.

Freezing behavior in the contextual fear task is considered to be a measurement of hypervigilant states^49^. Further, it is regulated by BNST activity^50,51^ and AIE exposure^20^ in male rodents. We induced freezing activity using low-intensity, unpredictable foot shocks^20^ (Fig. 4A). Although a vapor history*viral status ANOVA revealed no significant interaction or main effects (*p*’s > 0.05) on pre-shock freezing levels in female mice (Fig. 4B), there was a significant interaction effect in male mice [*F*(1,35) = 4.62, *p* < 0.05] without any significant main effects or differences between the groups (Fig. 4D). Change scores were used to account for these individual differences in basal freezing levels and intra-sex variability in behavior^5,47^. Freezing at all timepoints was adjusted for each individual mouse’s basal pre-shock freezing level (average freezing during the 30 s immediately preceding the shock). The fear acquisition was quantified as the change in freezing immediately post-shock (30 s), retention of contextual fear memory was quantified as the change in freezing during the initial 1 min re-exposure to the chamber 24 h following training, and extinction was quantified as the change in freezing after 8 min of re-exposure to the chamber with no shock presentation (Fig. 4A). Minute 8 was chosen because freezing levels begin to increase by minute 15 in this low-intensity foot shock task, possibly reflecting habituation to the chamber^20^.

In female mice, a vapor history*viral status*time point ANOVA revealed a main effect of time point [*F*(1.92,65.14) = 8.81, *p* < 0.001]. Given our previous findings of time point-specific effects^20^, we also completed vapor history*viral status ANOVAs for each individual time point. We observed no significant interaction or main effects on fear acquisition within the training session or freezing during initial contextual re-exposure (*p*’s > 0.05; Fig. 4C). However, there was a significant main effect of the virus on freezing at extinction minute 8 [*F*(1,37) = 5.31, *p* < 0.05], where GFP mice showed less freezing (Fig. 4C).

In male mice, a vapor history*viral status*time point ANOVA only revealed a main effect of time [*F*(2.00,69.89) = 3.15, *p* < 0.05]. At the post-shock point, a vapor history*viral status ANOVA revealed no significant interaction or main effect of virus (*p*’s > 0.05), but a significant effect of vapor history on fear acquisition within the training session [*F*(1,35) = 4.98, *p* < 0.05] (Fig. 4E). However, Sidak’s post-hoc analyses revealed that there was no significant effect of AIE-history within either viral status group (*p*’s > 0.05). No significant interaction or main effects were observed at the re-exposure or extinction minute 8 timepoints. These results support our previous finding that AIE-history enhances contextual fear acquisition within the training session in male mice^20^. Notably, this behavior was not affected by mGlu5 receptor knockdown in the BNST.

### Negative affect-like facets have minimal interdependency

Negative affective states represent a cluster of symptoms^6^, yet many preclinical studies are unable to investigate the relationship between these symptoms. Because most mice in the current study were run through all behavioral tasks, we were able to assess the individual relationships between basal anxiety (% center distance, NIH train latency), anhedonia (NIH test day latency), and hypervigilance (fear acquisition, re-exposure freezing, and extinction freezing). Detailed analyses revealed that vapor history and viral status had minimal influence on the relationships between these behavioral factors of negative affect, suggesting poor interdependence between components of negative affective-like states (see the Supplementary material and Supplemental Table 2 for more comprehensive analyses).

## Discussion

The current study replicates our previous findings that AIE history and stress in adulthood produce sex-specific behavioral outcomes in preclinical tasks that characterize negative affective-like states and are related to BNST dysregulation (summarized in Table 1). In female mice, AIE history and restraint stress increased latency to consume an appetitive reinforcer, indicating an enhanced anhedonic-like state that was independently increased by BNST-mGlu5 receptor knockdown. This notable finding points to specific effects of BNST mGlu5 signaling in regulating anhedonic behavior in female mice. Female mice with mGlu5 knockdown also displayed persistent freezing at the extinction time point compared to viral control mice in a manner that was not modulated by AIE. In males, AIE history enhanced fear acquisition within the training session but mGlu5 knockdown did not alter this response. However, mGlu5 knockdown did increase basal anxiety levels in male mice.

**Table 1:** Summary of findings.

	NIH test latency		Open field test		Fear conditioning	
	Air	AIE	Air	AIE	Air	AIE
*Female mice*						
GFP	Increased latency in AIE compared to air #, and increased latency in Cre compared to GFP		No change in % center distance for vapor history*viral status		(Control)	No change from control
Cre					mGlu5 knockdown enhanced freezing during extinction regardless of vapor treatment	
*Male mice*						
GFP	No change in NIH latency for vapor history*viral status		(Control)	No change from control	(Control)	Enhanced fear acquisition # regardless of viral status
cre			mGlu5 knockdown decreased % Center Distance (increased negative affective-like behavior)	No change from control	No change from control	

Summary of experimental findings. The pound sign (#) indicates replications of previous work.

### AIE produces distinct behavioral profiles mediated by BNST-mGlu5 receptors

Previous work using the NIH task has indicated that AIE history enhances latency to consume an appetitive reinforcer in female mice, but only following restraint stress^20^. In the current work, BNST-mGlu5 receptor knockdown increased the latency to consume on the NIH test day independent of AIE history in female mice but did not affect this behavior in male mice. This finding suggests that mGlu5 receptor signaling is required for maintaining baseline behavior, specifically in female mice, and that mGlu5 dysregulation increases anhedonic-like behavior regardless of prior insults. Interestingly, adult female mice with an AIE history show enhanced group 1 mGlu-mediated long-term depression (LTD) in the BNST following restraint stress, while male mice do not. This enhanced LTD is partially mediated by mGlu5 receptors^20^. In this previous work, overall mGlu5 levels were not altered by AIE in male or female mice^20^. However, these measurements were not able to determine whether the levels of mGlu5 receptors expressed in the plasma membrane were reduced. Alternatively, BNST-mGlu5 receptor knockdown could upregulate compensatory signaling (e.g., mGlu1 receptors or endocannabinoid signaling). Although the current studies are unable to differentiate between these two possibilities, they highlight the important role that mGlu5 signaling plays in regulating negative affect-like states in female mice. Further, it is possible that the results of these studies may have been different if the mGlu5 receptor knockdown surgery occurred after AIE treatment during late adolescence/early adulthood instead of prior to AIE during early adolescence. Due to the emergence of negative affective-like behaviors during adolescence^4^, these studies focused on concurrent mGlu5 receptor knockdown and vapor exposure during adolescent development to better capture relevant changes in mGlu5 signaling that may occur through the entirety of adolescence and play a role in alcohol-induced negative affect^26^.

In adult male mice, restraint stress enhances GluN2B-mediated signaling in the BNST of mice with an AIE history^25^. BNST-GluN2B receptors have been directly implicated in increased consumption latencies in the NIH task in male mice^45^. These results suggest that sexually divergent mechanisms identified in the BNST are directly related to distinct behavioral outcomes. Group 1 mGlu receptor regional expression levels or intracellular signaling cascades have been historically studied in male rodents. Sex differences in these signaling cascades have not been evaluated in the BNST. One aspect where known sex differences do exist is in convergence of estrogen and mGlu5 receptor intracellular signaling, whereby mGlu5 signaling can mediate estradiol-driven potentiation of psychostimulant-induced behaviors in female rodents (reviewed in ref. ^52^).

These distinct mechanisms are further supported by the subthreshold contextual fear results in males. This paradigm was used as a model of hypervigilance^48^, a facet of emotional behavior that is regulated by the BNST in humans and animals^21,50,51,53,54^. Although we observed increased post-shock freezing in AIE-exposed male mice, there was no influence of mGlu5 receptor knockdown on this behavior. As observed previously^20^, AIE history did not significantly alter fear acquisition within the training session in female mice. However, BNST-mGlu5 receptor knockdown did increase freezing in female mice at the extinction timepoint. It is unclear whether this represents persistent fear or is indicative of acclimation to the chamber. The latter appears to be the case in AIE-cre female mice, which showed relatively low levels of freezing at the re-exposure timepoint. This male-specific enhanced fear acquisition within the training session may also be indicative of increased BNST-GluN2B-mediated signaling^25^. Fyn kinase, which regulates GluN2B phosphorylation, has been shown to mediate contextual fear conditioning^55–57^. Previous work has indicated that high alcohol-preferring mice exposed to cued fear conditioning and allowed free access to alcohol show enhanced hypervigilance in the fear-potentiated startle task, regardless of sex. However, fear conditioning only caused immediate increases in alcohol intake in male mice^58^. Another model using unpredictable chronic mild stress showed that an alcohol history plus stress increased binge drinking in male, but not female, mice^59^. Although these studies fundamentally differ from the current model, they may suggest that the hypervigilant state in males driven by adolescent alcohol exposure may fuel increased alcohol intake in adulthood.

Sex-dependent effects were also observed in the open field, where BNST-mGlu5 knockdown increased anxiety-like behavior in air-cre, but not AIE-cre, mice. Previous work observing the effects of adolescent alcohol exposure on anxiety indices in adulthood has shown contradictory effects^10–12,20^. Further, the handful of studies observing the role of mGlu5 receptors in adult anxiety-like activity following adolescent alcohol exposure have not demonstrated clear effects of intervention^12,60^. It could be speculated that BNST-mGlu5 knockdown reduced gating of a male-specific neurocircuit involved in the expression of anxiety-like activity. For example, the kappa opioid-mediated basolateral amygdala to BNST projection regulates the unconditioned anxiety-like activity and mediates excitatory activity in the BNST^61^. Whether such circuits are male-specific and mediated by alcohol exposure is unknown.

These sex-specific enhancements of negative affect-like behavior in mGlu5 knockdown mice are surprising given the role of mGlu5 antagonists and negative allosteric modulators in treating depression- and anxiety-like behavior in rodents, as well as the observed behavioral phenotypes of global mGlu5 knockout (KO) animal models. There are numerous possible explanations for the discrepancy between the current results and this literature. First, mGlu5 receptors in the current study are not abolished but are closer to a 60% reduction (see Fig. 1B). There is evidence in some studies that mGlu5 heterozygous (HET) and KO mice show divergent phenotypes from their wild-type counterparts. For example, HET mice show an enhancement of operant sensation seeking, whereas KO mice show a reduction in their behavioral response^62^. The mice in the current study may be more similar to the HET mice receptor levels versus complete KO. However, divergence in HET/KO phenotypes has not generally been seen in studies of generalized anxiety, although to our knowledge NIH has not been assessed. The second potential explanation could be due to the site- and circuit-level roles of the BNST in anxiety and anhedonic behaviors. Optogenetic studies have shown that the behavioral valence outcome of BNST modulation depends on the subnuclei activated^63^. While Cre expression in this study (and thus mGlu5 receptor knockdown) does not seem to be confined to a particular subnuclei, it is possible that mGlu5 receptor gating of excitatory transmission in this region may have divergent actions. Alternatively, mGlu5 knockdown throughout the BNST may change the activity of specific circuits involved in negative affect-like behaviors. The BNST is a highly interconnected brain region, with reciprocal connections to other regions known to be involved in negative affect, including the cortex, amygdala, and ventral tegmental area^64–66^. mGlu5 receptor knockdown may disrupt LTD in the BNST, particularly in female mice following stress^20,67,68^. Disrupted LTD in the BNST could disturb the balance of excitatory and inhibitory transmission throughout these circuits, resulting in disrupted behavioral outcomes.

### Translational relevance

These findings have robust clinical relevance for investigating underlying mechanisms of the sexually dimorphic risks associated with problematic alcohol use. The NIH task is a model of anhedonia, a classic symptom of depression^44^. Notably, depressive symptoms are stronger drivers of initial and continued alcohol use in young girls and women compared to young boys and men^8^. A recent Phase II clinical trial reported that the mGlu5 negative allosteric modulator basimglurant did not significantly reduce clinician-rated symptoms of MDD in a large sample of individuals without recent alcohol abuse. However, the high dose group (69.4% female) showed significant improvements in self-reported symptoms of depression^36^. In addition, recent genome-wide association studies consistently identify *GRM5* as a significantly changed gene in patients with MDD^69–71^. Paired with the current findings, these results suggest that mGluR5 receptors present an encouraging druggable target and that targeting mGlu5 receptors may alleviate depressive states in women that could proceed problematic alcohol use.

Freezing to low-intensity, unpredictable foot shocks is indicative of a hypervigilant state^49^. Hypervigilance is a distinct facet of the hyperarousal criteria in post-traumatic stress disorder (PTSD)^72^. Although hypervigilance is characteristic of many disorders, like PTSD and generalized anxiety, it is specifically enhanced by unpredictable aversive events in individuals with PTSD^73^. Within PTSD, hypervigilance is a strong predictor of experiencing other PTSD-related outcomes^74^, which may be associated with problematic alcohol use. Women are more likely to be diagnosed with PTSD than men, but men may be more likely to present with a comorbid AUD^75^. In predominantly male veteran populations with comorbid AUD, hyperarousal is associated with intrinsic drinking motives and poor adherence to an online PTSD treatment program^76,77^. Although comorbid PTSD and AUD are strongly associated with emotional regulation facets of PTSD in women, this does not include hyperarousal^78^, similar to the findings in our preclinical model. Our findings may also indicate that adolescent alcohol exposure heightens the risk of developing PTSD-related symptoms.

## Conclusion

Preclinical models with high face validity are necessary to develop treatments for AUDs and comorbid disorders. Although many studies have found that reducing mGlu5 activity alleviates alcohol-induced negative affective states, these studies have focused on males and have not widely included models of dependence. The current findings indicate that AIE produces sexually divergent behavioral phenotypes in adulthood that are unmasked by stress and reflect the human condition. The underlying symptoms and mechanisms of these factors also appear to be sexually divergent, with mGlu5 receptors playing a large role in depression-related risks in female mice. Notably, these behavioral facets showed poor interdependency, which is further indicative of distinct mechanisms. Overall, these findings support the use of mGlu5 receptors as a treatment target to alleviate negative affective states that may precipitate problematic drinking behavior.

## Supplementary information

Supplemental Information
