# Supplementary material for: BNST specific mGlu5 receptor knockdown regulates sex-dependent expression of negative affect produced by adolescent ethanol exposure and adult stress
Source: Transl Psychiatry. 2021 Mar 17;11:178. doi: 10.1038/s41398-021-01285-y (PMC7969933; doi:10.1038/s41398-021-01285-y)
Supplement: Supplementary file 1 — Supplemental Information [file 41398_2021_1285_MOESM1_ESM.docx]

**Supplemental**

Supplemental Table 1: Number of mice used

| **Female** | **Beginning group size** | **Lost following surgery** | **Off-target surgery** | **Used for behavior** | **Run through all tasks** | **Open field/fear only** | **NIH only** |
| --- | --- | --- | --- | --- | --- | --- | --- |
| Air-GFP | **13** | 1 | 1 | **11** | 8 | -- | 3 |
| Air-cre | **13** | -- | 1 | **12** | 10 | -- | 2 |
| AIE-GFP | **13** | 1 | -- | **12** | 10 | -- | 2 |
| AIE-Cre | **12** | 1 | -- | **11** | 8 | -- | 3 |
| **Male** |  |  |  |  |  |  |  |
| Air-GFP | **13** | 2 | -- | **11** | 11 | -- | -- |
| Air-cre | **11** | 1 | 1 | **9** | 8 | -- | 1 |
| AIE-GFP | **11** | -- | -- | **11** | 8 | 3 | -- |
| AIE-Cre | **9** | -- | -- | **9** | 7 | 2 | -- |
| **Total** | **95** |  |  | **86** |  |  |  |

First, simple linear regression analyses were run comparing each pair of behavioral outcomes within vapor history*viral status for each sex. The slopes of the lines were statistically compared to observe any significant vapor history*viral status influence on behavioral relationships. Correlations including all animals ran within a task were also run to observe overall behavioral relationships (Supplemental Table 2). In female mice, vapor history*viral status did not significantly alter the relationship between any pair of behavioral tasks, as indicated by no significantly different slopes (*p*’s > 0.05). Overall relationships indicated that freezing during the contextual fear task was highly correlated across all timepoints. Greater fear acquisition within the training session, indicating a larger change in freezing between the 30 s immediately pre- and post-shock, was positively correlated with freezing during the initial re-exposure and freezing at minute 8 of the extinction session (*p*’s < 0.001). Further, freezing during re-exposure and at minute 8 of the extinction period were positively correlated (*p* < 0.001). These results indicate that female mice that showed the greatest fear acquisition within the training session also showed persistent contextual freezing upon re-exposure, regardless of vapor or viral status. There was also a significant overall relationship between fear acquisition and % center distance, where increased freezing post shock was related to more time spent in the center of the open field prior to shock (*p* < 0.05). In other words, ​increased freezing immediately following the shocks was associated with lower levels of basal anxiety-like activity in the open field.

In male mice, vapor history*viral status mediated the relationships between NIH train latency and a number of metrics, including % center distance [F(3,23) = 4.63, *p* < 0.05], re-exposure freezing [F(3,23) = 4.84, *p* < 0.01], and NIH test latency [F(3,23) = 3.54, *p* < 0.05]. In air-GFP mice, a higher NIH train latency was associated with less % center distance in the open field [r(9) = -0.727, *p* < 0.05)], indicating a relationship between basal measures of anxiety-like activity in this control group. The air-GFP group also had the only significant correlation between NIH train latency and re-exposure freezing, where less freezing was associated with a greater train latency [r(8) = -0.758, *p* < 0.05], indicating an inverse relationship between basal anxiety and contextual fear. The relationship between NIH train and test latencies was restricted to mice with BNST-mGlu5 receptor knockdown. In the air-cre mice, a higher NIH train latency was associated with a lower consumption latency on the test day [r(7) = -0.862, *p* < 0.05]. Conversely, AIE-cre mice with increased NIH train latency showed an increased test day latency [r(8) = 0.854, *p* < 0.01].

Similar to female mice, male mice showed overall significant relationships between fear acquisition within the training session, re-exposure freezing, and extinction freezing (*p*’s < 0.001), where increased freezing at one time point was associated with increased freezing at all other time points. Overall relationships indicated that freezing during all timepoints of the contextual fear task was highly correlated in male and female mice. Vapor history and viral status did not significantly alter the relationship between any pair of behavioral tasks in female mice (Supplemental Table 2). In male mice, vapor history and viral status produced a significant relationship between NIH train latency and % center distance, re-exposure freezing, and NIH test latency in air-GFP control mice indicating a relationship between basal measures of anxiety-like activity in this control group. Overall, there was poor interdependence between components of negative affective-like states.

**Supplemental Table 2** Behavioral correlations

|  | **% Center Distance** | | **Fear**  **Acquisition** | | **Re-exposure Freezing** | | **Extinction Freezing** | | **NIH Train Latency** | | **NIH Test Latency** | |
| --- | --- | --- | --- | --- | --- | --- | --- | --- | --- | --- | --- | --- |
| **Females** | r | *p* | r | *p* | r | *p* | r | *p* | r | *p* | r | *p* |
| **% Center Distance** | 1 | - | 0.382 | **< 0.05** | 0.225 | > 0.05 | 0.105 | > 0.05 | 0.217 | > 0.05 | -0.286 | > 0.05 |
| **Fear**  **Acquisition** |  |  | 1 | - | 0.580 | **< 0.001** | 0.623 | **< 0.001** | -0.212 | > 0.05 | -0.186 | > 0.05 |
| **Re-exposure Freezing** |  |  |  |  | 1 | - | 0.620 | **< 0.001** | -0.324 | >  0.05 | 0.049 | > 0.05 |
| **Extinction Freezing** |  |  |  |  |  |  | 1 | - | -0.205 | > 0.05 | -0.027 | > 0.05 |
| **NIH Train Latency** |  |  |  |  |  |  |  |  | 1 | - | -0.320 | = 0.09 |
| **NIH Test Latency** |  |  |  |  |  |  |  |  |  |  | 1 | - |
|  | **% Center Distance** | | **Fear**  **Acquisition** | | **Re-exposure Freezing** | | **Extinction Freezing** | | **NIH Train Latency** | | **NIH Test Latency** | |
| **Males** | r | *p* | r | *p* | r | *p* | r | *p* | r | *p* | r | *p* |
| **% Center Distance** | 1 | - | 0.2355 | > 0.05 | 0.234 | > 0.05 | 0.236 | > 0.05 | 0.163  * | > 0.05 | 0.193 | > 0.05 |
| **Fear**  **Acquisition** |  |  | 1 | - | 0.530 | **< 0.001** | 0.540 | **< 0.001** | -0.157 | > 0.05 | 0.058 | > 0.05 |
| **Re-exposure Freezing** |  |  |  |  | 1 | - | 0.508 | **< 0.001** | -0.102 | > 0.05 | 0.159 | > 0.05 |
| **Extinction Freezing** |  |  |  |  |  |  | 1 | - | -0.151 | > 0.05 | 0.125 | > 0.05 |
| **NIH Train Latency** |  |  |  |  |  |  |  |  | 1 | - | -0.249  * | > 0.05 |
| **NIH Test Latency** |  |  |  |  |  |  |  |  |  |  | 1 | - |

Asterisk (*) denotes simple linear regression analyses that indicated significantly different slopes between vapor history*viral status groups. Correlations assessing NIH behaviors did not include non-consumers (n’s = 30 for females, 31 for males). Correlations without NIH behaviors included all mice tested (n’s = 37 for females, 39 for males).
